# Bubble dynamics and speed of jets for needle-free injections produced by thermocavitation

**DOI:** 10.1117/1.JBO.28.7.075004

**Published:** 2023-07-21

**Authors:** Nancy Elizabeth González-Sierra, José Manuel Perez-Corte, Juan Pablo Padilla-Martinez, Samuel Cruz-Vanegas, Silvio Bonfadini, Filippo Storti, Luigino Criante, Rubén Ramos-García

**Affiliations:** a Instituto Nacional de Astrofísica, Óptica y Electrónica, Coordinación de Óptica, Puebla, México; b Benemérita Universidad Autónoma de Puebla, Instituto de Ciencias, Puebla, México; c Istituto Italiano di Tecnologia, Center for Nano Science and Technology, Milano, Italy; d Politecnico di Milano, Department of Physics, Milano, Italy

**Keywords:** needle-free injection, thermocavitation, continuous wave laser, bubble dynamics, liquid jet, chemical etching

## Abstract

**Significance:**

The number of injections administered has increased dramatically worldwide due to vaccination campaigns following the COVID-19 pandemic, creating a problem of disposing of syringes and needles. Accidental needle sticks occur among medical and cleaning staff, exposing them to highly contagious diseases, such as hepatitis and human immunodeficiency virus. In addition, needle phobia may prevent adequate treatment. To overcome these problems, we propose a needle-free injector based on thermocavitation.

**Aim:**

Experimentally study the dynamics of vapor bubbles produced by thermocavitation inside a fully buried 3D fused silica chamber and the resulting high-speed jets emerging through a small nozzle made at the top of it. The injected volume can range from ∼0.1 to 2  μL per shot. We also demonstrate that these jets have the ability to penetrate agar skin phantoms and *ex-vivo* porcine skin.

**Approach:**

Through the use of a high-speed camera, the dynamics of liquid jets ejected from a microfluidic device were studied. Thermocavitation bubbles are generated by a continuous wave laser (1064 nm). The 3D chamber was fabricated by ultra-short pulse laser-assisted chemical etching. Penetration tests are conducted using agar gels (1%, 1.25%, 1.5%, 1.75%, and 2% concentrations) and porcine tissue as a model for human skin.

**Result:**

High-speed camera video analysis showed that the average maximum bubble wall speed is about 10 to 25 m/s for almost any combination of pump laser parameters; however, a clever design of the chamber and nozzle enables one to obtain jets with an average speed of ∼70  m/s. The expelled volume per shot (0.1 to 2  μl) can be controlled by the pump laser intensity. Our injector can deliver up to 20 shots before chamber refill. Penetration of jets into agar of different concentrations and *ex-vivo* porcine skin is demonstrated.

**Conclusions:**

The needle-free injectors based on thermocavitation may hold promise for commercial development, due to their cost and compactness.

## 1 Introduction

Syringe-based drug delivery has been the most common and efficient method for more than a century.^1^ With the advent of the COVID-19 pandemic, more than 10 billion vaccines have been administered worldwide. This number increases by at least an order of magnitude when other regularly administered vaccines are included. As a result, tens of billions of needles are produced and wasted each year. The disposal of needles requires special procedures to avoid punctures, yet accidental punctures occur among medical and cleaning staff, exposing them to highly contagious diseases such as hepatitis and human immunodeficiency virus.^2^^,^^3^ In addition, needle phobia may prevent adequate treatment.^4^ To overcome these problems, needle-free injectors were developed in the 1970s,^5^ but several reasons prevented their massive use: the possibility of cross-contamination, their cost, the complexity of use, and the instability of the jet, which tends to break, causing bruising and even bleeding.^6^^–^^8^

Most needle-free injectors consist of three parts: (a) the energy source to propel the drug, (b) a chamber to contain the drug to be expelled, and (c) a nozzle from which the liquid jet is expelled. Commercial injectors use either compressed air,^9^ loaded springs,^10^ piezo actuators, or electrical discharge as the actuation mechanism,^11^^–^^13^ to produce a thin but powerful enough jet to penetrate the skin. Recently, laser-induced jets have been shown to be more stable than those produced by electromechanical means.^14^^,^^15^ In addition, the presence of a meniscus near the nozzle has resulted in highly focused jets achieving speeds of up to 850 m/s and penetration of several millimeters into the skin.^16^ To achieve this speed, a laser beam from a nanosecond pulsed laser is focused into water (or any other transparent liquid) to create a plasma by nonlinear absorption;^17^^,^^18^ the plasma heats the water, creating a vapor bubble that rapidly expands and collapses, violently emitting pressure waves of a few GPa.^19^ However, these high-speed jets typically have a relatively small diameter (<100  μm), which limits the volume of drug injected.^20^

Recently, a novel mechanism of bubble generation using continuous-wave lasers to produce high-speed jets has been reported.^21^ Here, a low-to-medium power continuous wave (CW) laser is focused into a highly absorbing solution to superheat the liquid and produce rapidly expanding bubbles.^22^ Bubble formation by CW lasers is known as thermocavitation. In this mechanism, the process of bubble formation is a linear phenomenon compared to pulsed lasers, but the speed of the jets is not so different from those obtained with pulsed lasers.^23^^,^^24^ Thermocavitation-based injectors have been shown to be effective for drug delivery in the superficial layers of the skin (epidermis and dermis) and have been proposed for therapeutic or cosmetic applications due to the small volume delivered^25^^,^^26^ compared to electromechanical injectors.^27^^,^^28^

In this work, we experimentally studied the dynamics of bubbles generated by thermocavitation inside a transparent chamber and the resulting jets generated by their expansion after passing through a readily adaptable nozzle (in our case, 200  μm internal diameter). Using a high-speed camera, we found that the maximum average speed of the bubble wall is about 10 to 25 m/s for almost any combination of laser parameters, suggesting that the jet speed is limited by the bubble dynamics; however, using a convenient chamber design, a jet average speed of ∼70  m/s was obtained. Jet penetration into agar of various concentrations is demonstrated and our results indicate an average penetration of ∼1  mm in the hardest agar (2%). The volume of a single jet is increased to ∼2  μl with an impact jet power of ∼7  W, far exceeding the threshold power to break the skin. It is worth noting that this achieved capacity exceeds typical volumes (50 nl^29^ in previous systems based on thermocavitation^21^) by more than an order of magnitude. Finally, a comparison with other needle-free injectors including commercially available mechanically and electromechanically is presented. These injectors have been used extensively in the medical field for delivering vaccines and other medications. These injectors are relatively affordable and simple to operate, making them a popular choice for healthcare providers. However, they can be painful and uncomfortable for patients, and may also require regular maintenance. Thermocavitation-based injectors, as discussed in our paper, offer the potential for low-cost and compact designs. However, further research is needed to optimize these devices and ensure their safety and efficacy.

## 2 Chamber Design and Fabrication

Most of the work using pulsed lasers to generate high-speed jets uses devices based on capillary tubes, but these are not practical for real applications because they are fragile, empty after each shot and, most importantly, deliver tiny amounts of drugs. More interesting, but less common due to their design and manufacturing complexity, are injectors consisting of a chamber containing the liquid and a nozzle from which the liquid is ejected. Park et al.^30^ were the first to fabricate a chamber divided in two by a flexible membrane to separate the cavitating liquid from the drug to be injected. Following their example, we numerically optimized the cavity design to maximize the jet speed^31^^,^^32^ and 3D printed several cavity designs, but due to the limited spatial resolution (typically ∼16  μm axial and >30  μm lateral) of the 3D printer used, excessive roughness remaining in the outlet channel and nozzle significantly affected the stability and quality of the jets. To reduce the roughness, we fabricated cavities in glass by simple chemical etching, but the resulting cavities were too small and emptied each time a jet was fired, limiting the practical development of the injector.^21^^,^^33^

To advance the chamber injector, a significant improvement in the manufacturing process is required to ensure robustness, rapid prototyping, and freedom in the design of the new devices in terms of shapes, geometries, and nozzle sizes. In this way, the performance of the final device can be easily optimized according to different design parameters. For these reasons, the devices are fabricated buried in fused silica substrates using the femtosecond laser irradiation followed by chemical etching (FLICE) technique.^34^^–^^36^ FLICE is a two-step fabrication process: (a) permanent 3D high-resolution modification of the physical and chemical properties of the substrate by femtosecond laser irradiation, (b) subsequent selective removal of the laser-modified material by wet chemical etching (typically HF or KOH).

**Fig. 1:**
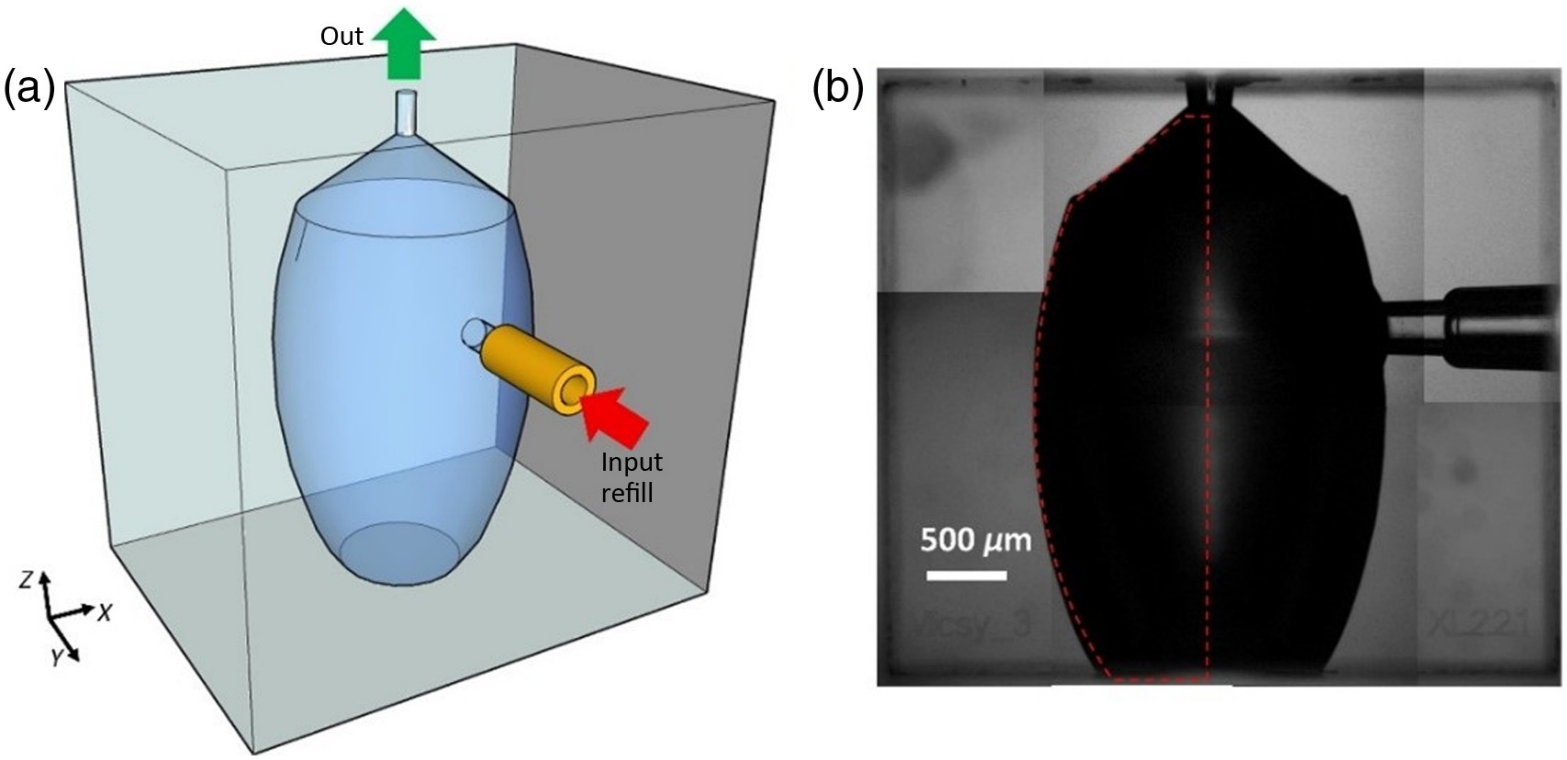
(a) Sketch of the needle-free injector microfluidic device in continuous flow. (b) Image of the buried device. The volume of internal material removed is about of $9\;{\mathrm{mm}}^{3}$. The “monolithic chip” fabrication method, rather than welding two halves together, ensures that high internal pressure is achieved without breakage or leakage.

The high pressure and stress generated in the volume of a tightly focused femtosecond laser beam first cause a reduction in the Si-(O)-Si bridging angle^37^ and then induces a permanent and localized periodic redistribution of material density in the bulk of the fused silica.^38^ The generation of these nanogratings plays a key role in promoting access of the etchant solution to the material to be removed, resulting in a large difference in etch rate between laser-modified and pristine material. Selective material removal is thus possible, allowing the creation of hollow 3D structures that are monolithically buried in the substrate. Thanks to this innovative, maskless, direct fabrication technique, we were able to fabricate a large-volume chamber in glass that allows up to 20 shots before refilling (see Fig. 1). In addition, the microfluidic configuration of the device allows continuous and automatic refilling of the cavity through a cylindrical side port using an infusion pump. The nozzle (200  μm inner diameter) is designed to increase the velocity of the jet, with a conical shape ending in a 200  μm long cylindrical channel from which the jet is expelled.

Our micromachining setup consists of an amplified Yb:KGW femtosecond laser system (Pharos, Light Conversion), with a fundamental emission wavelength of 1030 nm. Several parameters, including pulse duration (240 fs to 10 ps), repetition rate (1 kHz to 1 Mhz), pulse energy (up to ∼0.2  mJ), and average power (up to ∼10  W), are user-controlled. The generation of ultrashort and high-power pulses is implemented by a standard chirped-pulse amplification mechanism. Then, through an electro-optical shutter, the repetition rate at which the laser pulses leave the system can be selected without changing the characteristics of the laser cavity. An external harmonic generator (HIRO, light conversion) allows generation of the second harmonic (515 nm) typically used for this kind of chip fabrication. The laser light is focused statically, through an objective (50×, Mitutoyo), inside the substrate. Computer-controlled, three-axis motion stages (ABL-1000, Aerotech, Pittsburgh, Pennsylvania, United States) interfaced by computer-aided design (CAD)-based software (ScaBase, Altechna, Vilnius, Lithuania) with an integrated acousto-optic modulator were used to translate the sample relative to the laser irradiation beam. The syringe chamber volume was fully irradiated with 600 nJ/pulse (λ=515  nm, P=300  mW, repetition rate = 500 kHz), whereas the substrate was moved at a speed of 1 mm/s. Because the volume of material to be removed is a challenge for a monolithic chip (about $9\;{\mathrm{mm}}^{3}$), the writing trajectories and polarization were designed to reduce etching times. The chemical treatment was performed by immersing the sample in an ultrasonic bath of 20% aqueous hydrofluoric acid solution for 10 h.

## 3 Experimental Setup

**Fig. 2:**
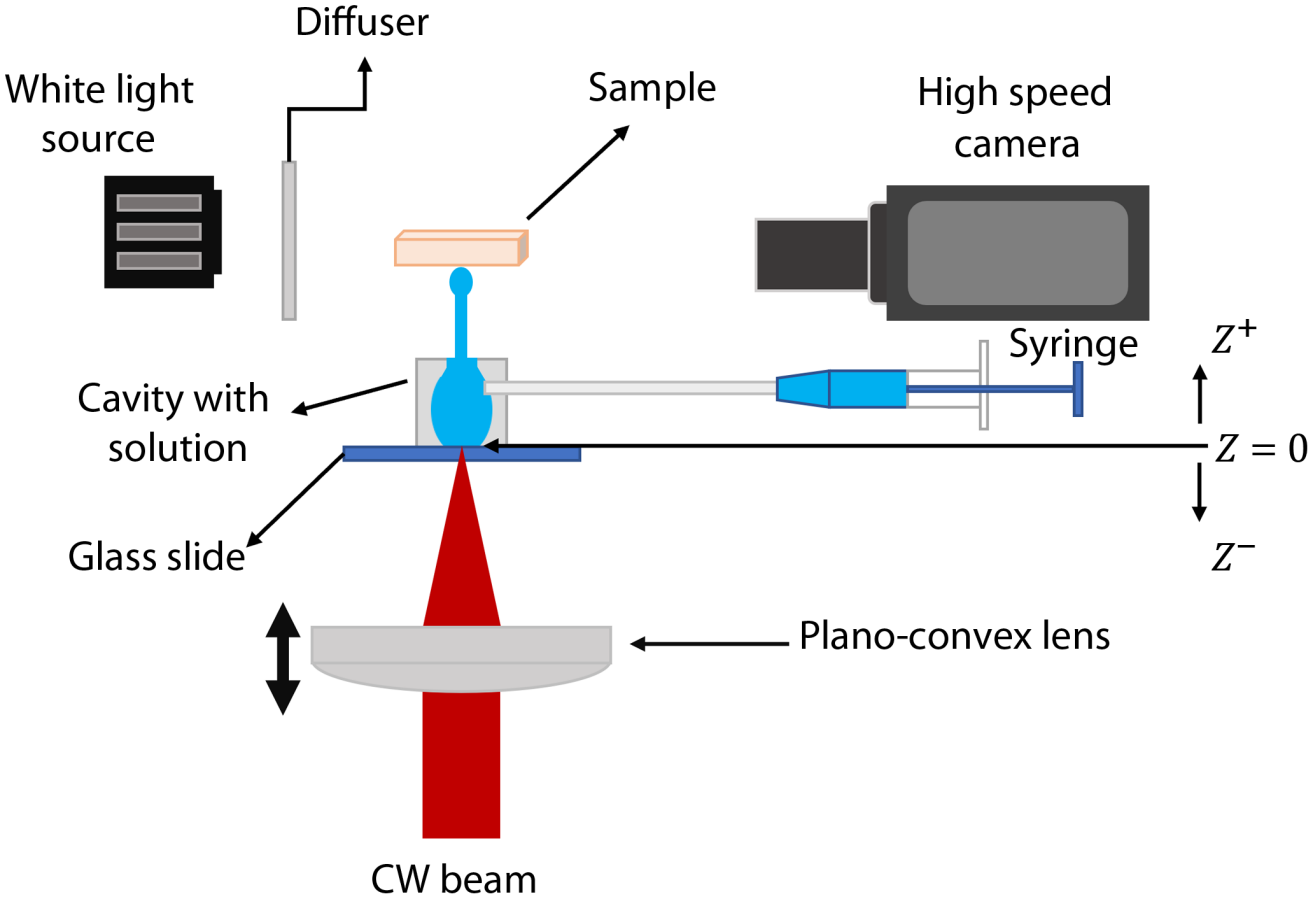
Experimental setup for the generation of bubbles and high-speed jets. The laser beam is focused on the glass-liquid interface of the chamber. Bubble and jet dynamics are captured by a high-speed camera.

Figure 2 shows a schematic of the experimental setup. A collimated beam from a CW fiber-coupled laser (IPG Photonics Model YLR-5-1064-LP operating at 1064 nm, beam diameter 1.5 mm, $M^{2\;}=1.05$, and a maximum power of 5 W) is focused on the lower glass-liquid interface of the chamber using a 5 cm focal length lens (Thorlabs LA1213-BK7). The chamber is laterally illuminated by a high-power halogen lamp to visualize and record the bubble dynamics, which are captured by a high-speed camera (Phantom v311). The chamber was filled with a saturated solution of copper nitrate $\mathrm{Cu}({\mathrm{NO}}_{3}{)}_{2}$ (13.78 gr of $\mathrm{Cu}({\mathrm{NO}}_{3}{)}_{2}$ dissolved in 10 ml of deionized water). The absorption coefficient of the solution at the operating wavelength is $∼130\;{\mathrm{cm}}^{-1}$, which means that the light is essentially absorbed near the face entrance (light penetration depth ∼75  μm). Skin phantoms or *ex-vivo* porcine skin was placed 5 mm above the jet exit and the dynamics of the jet penetration were also analyzed. Thermocavitation is a self-organizing phenomenon, which means that when the laser is on continuously, the bubble generation (and collapse) occurs in a quasi-periodic manner,^22^ allowing the cavitation frequency (defined as the number of bubbles generated per second) to be measured. To generate a single cavitation event, an electronic shutter was placed at the laser output to control the illumination time.^22^

The beam waist radius at the focal point of the lens ($\omega_{0}∼22\;\mu m$) and the corresponding Rayleigh distance ($z_{R}∼1.5\;\mathrm{mm}$) were calculated using the equations for the transmission of Gaussian beams through a thin lens.^39^ Displacing the lens holder changes the focal position inside (z>0) or outside (z<0) the chamber. This parameter changes the beam spot and therefore the light intensity at the glass-liquid interface. For different distances z, the beam waist is given by $\omega (z)=\omega_{0}(1+(z/z_{R}{)}^{2}{)}^{1/2}$.^39^ In our experiments, the focal point position z was varied from 10 mm [ω (10 mm) ∼152.2  μm] to 34 mm [ω (34 mm) ∼510.5  μm] at z=4  mm intervals. For z<10  mm, the cavitation frequency is the highest, but the radius of the bubbles is the smallest. This trigger only a small perturbation on the liquid near the nozzle not enough to generate liquid jets.

Skin phantoms were prepared using agar (SIGMA-ALDRICH 9002-18-0) at different agar concentrations (1%, 1.25%, 1.5%, 1.75%, and 2% per volume). Agar samples were prepared on a weight percentage basis, e.g., for a 2% concentration, 2 g of agar powder was dissolved in 100 ml of distilled water. For each sample, the initial water content was divided into two equal parts. The agar was added to one half while the other half was boiled. Once the second half was boiled, the first half with the dissolved agar was added and the sample was heated until no air bubbles formed in the mixture. After cooling at room temperature for 20 min, pieces of 1.5  cm×1.5  cm×1  cm were cut. A homemade system was used to measure the Young’s modulus for our samples obtaining the following values: 34, 51, 59, 86, and 113 kPa for the agar samples with concentrations of 1%, 1.25%, 1.5%, 1.75%, and 2%, respectively, which agree well with previous measurements.^40^^,^^41^ Freshly prepared samples were placed above the exit nozzle and jets of different speeds were fired. The penetration distance was measured by means of video analysis from the high-speed camera.

## 4 Results and Discussions

### 4.1 Thermocavitation Bubble Generation

The absorption of light in the highly absorbent solution rapidly increases its temperature beyond the boiling point without doing so. Around the spinodal limit ($∼300^{{}^{\circ}}C$ for pure water), the liquid reaches a metastable state (superheated water) where any perturbation on the liquid density produces an explosive liquid-to-vapor phase transition generating a fast-expanding vapor bubble.^22^ Given the high absorption of the liquid, the bubble is basically generated at the glass-liquid interface and it evolves attached to the glass substrate taking a hemispherical shape.^22^ The radius of the bubble depends on the intensity of the laser at the glass-solution interface. At high intensity, the bubbles are small since the rate of heating is so high that the spinodal limit is achieved in a time scale lower or comparable to the diffusion time producing smaller bubbles. On the contrary, at low intensity, the rate of heating is lower than the heat diffusion time producing larger bubbles.^22^^,^^42^ Thermocavitation is attractive for needle-free applications because the size and periodicity of the bubble can be controlled with the light intensity, in terms of needle-free injectors, it means, that the delivered volume and number of shots per second are light-controlled.

### 4.2 Bubble Dynamics and Jet Speeds

**Fig. 3:**
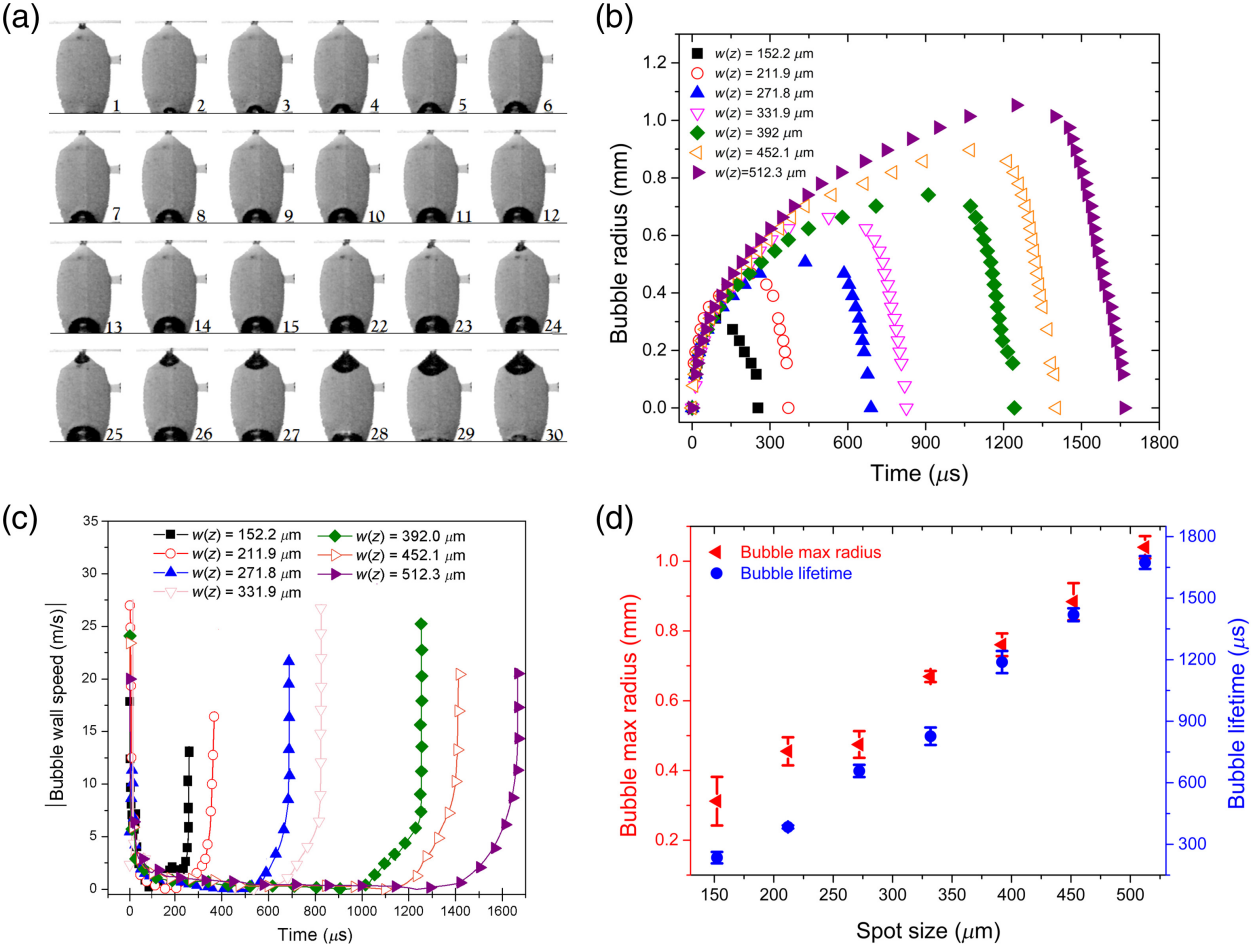
(a) Bubble formation and collapse inside the transparent chamber for a laser power of 590 mW, z=23  mm, and a spot size of $\omega_{z}=346.9\;\mu m$. Each frame is taken 37  μs apart and the elapsed time is obtained by multiplying the number of frames by the time interval between frames. (b) Bubble radius dynamics for different spot sizes. (c) Absolute value of the bubble wall speed. The lines are guide to eye only. d) Maximum bubble radius and bubble lifetime as a function of the laser spot size. The laser power used for (b), (c), and (d) was ~1 W.

When sufficiently large bubbles are generated in a chamber with a small aperture, the bubble expansion forces the liquid out of the chamber. This is shown in Fig. 3(a) where snapshots of the bubble dynamics are displayed every 37  μs but the capture frame rate was much higher (110,000 fps), with the beam focused inside the solution (z=23  mm, ω=346.9  μm, and power of P=590  mW). In frame 22, the bubble reaches its maximum radius and then begins to collapse afterward. In contrast to bubbles generated away from the container walls (using short-pulsed lasers in transparent solutions), up to 10 rebounds have been reported.^43^ In thermocavitation, most of the bubble energy is dissipated in the first collapse as a strong pressure wave and only a small bubble rebound is observed (frame 30), however, its size is not sufficient to expel liquid from the chamber producing only a small perturbation on the liquid-air interface inside the chamber. During the bubble expansion phase, the jet is generated (not shown). As the bubble begins to collapse a reentrant pressure causes the air enters the chamber (the dark region near the exit channel) as shown in frame 25. The volume of the ejected liquid is equal to the volume of the missing liquid as shown in frame 30. Figure 3(b) shows the bubble dynamics for different focusing positions inside the chamber. Note that the bubbles are smaller at higher intensities (smaller spot size) and increase as the intensity decreases (large spot size), as expected for thermocavitation bubbles. Note that the bubble collapse phase is much faster than the expansion one. Upon collapse, a strong pressure wave (∼2 to 3 MPa measured 4 mm away from the bubble collapse) is emitted.^42^ In fact, it has recently been demonstrated that this pressure wave can be used to eject a jet if the chamber is properly designed to focus the pressure wave near the exit channel.^22^^,^^25^

The bubble wall speed was calculated by taking the derivative of the position versus time. As can be seen in Fig. 3(c), the bubble wall speed is maximal immediately after bubble formation, then it goes to zero and finally increases again. Note that the bubble wall speed during expansion is approximately the same (∼10 to 25 m/s), regardless of the intensity. This means that the jet speed is basically determined by the bubble dynamics, which in turn is controlled by the liquid-to-vapor phase transition rate. Interestingly, the bubble wall speed produced by short-pulsed lasers (ps and ns) is comparable (by a factor of 2 to 3) to that produced by thermocavitation at the same time scale.^44^^,^^45^ Certainly, the bubble wall speed produced by pulsed lasers on short time scales (ns) could be very fast (∼2450m/s) but decreases rapidly to 100 to 300 m/s within 150 ns after plasma formation.^45^^–^^47^ From the video analysis, the bubble radius versus time was extracted showing that the bubble lifetime increases from ∼250  μs for the smaller bubbles (∼0.3  mm radius) up to ∼1700  μs for the largest bubbles (∼1  mm radius) as shown in Fig. 3(d). The maximum diameter of the bubbles is also comparable to those produced by pulsed lasers; therefore, CW laser-based injectors are a cheap and competitive option to pulsed laser-based injectors.

**Fig. 4:**
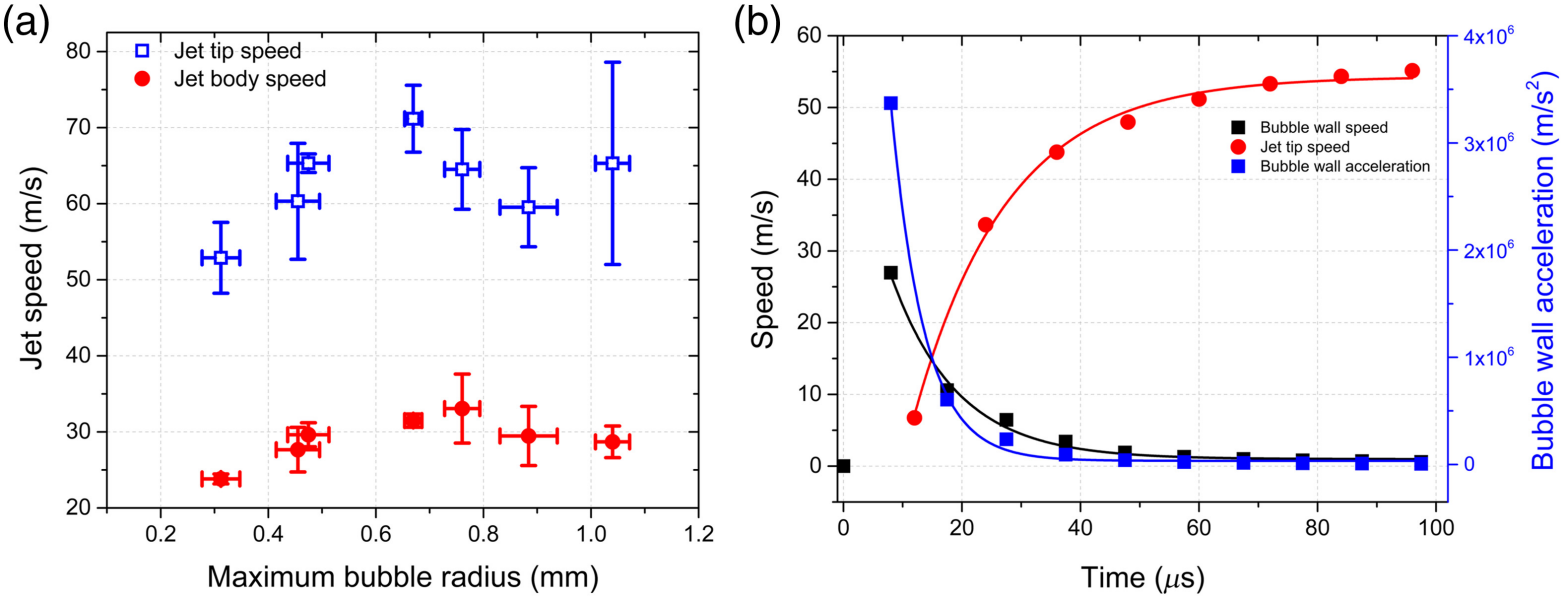
(a) Speed of the jet tip and body versus the maximum bubble radius. (b) Typical bubble wall (black squares) and jet speed (red circles) versus time. The continuous lines represent fits to an exponential decay and growth, respectively. The time constants are 11 and 15  μs, respectively. The blue squares represent the bubble wall acceleration and the continuous line a fit to an exponential decay function.

As mentioned above, the jet is generated during the expansion of the bubble, and its velocity is apparently determined by the expansion speed of the bubble wall. We found that the average jet speed ranges from 56 to 70 m/s with a large standard deviation [see Fig. 4(a)], which is characteristic of thermocavitation bubbles. Each experimental point represents an average of 15 shots. On average, the speed increases from ∼55 to ∼70  m/s as the intensity decreases (beam spot size increases) but appears to saturate around an average speed of ∼70  m/s. As the jet travels through air, it becomes unstable and eventually breaks up.^48^^,^^49^ The jet tip travels almost two times faster than the body jet.

Figure 4(b) provides a glimpse into the behavior of the bubble wall and jet speed as a function of time revealing the rapid acceleration and subsequent dynamics that drive the formation and ejection of the jet obtained with the following parameters: optical power of 1 W, z=14  mm, ω=212  μm, and frame rate of 110,000 fps. These conditions correspond to the second smallest bubble shown in Fig. 3(b). The continuous lines represent exponential decay ($Ae^{-t/t_{1}}$) and growth [$A(1-e^{-t/t_{2}})$] fits of the bubble wall speed and jet speed with time constants of $t_{1}∼11\;\mu s$ and $t_{2}∼15\;\mu s$, respectively.

The most striking observation is that the bubble wall achieves its maximum speed of ∼27  m/s within just one frame as can be seen in Fig. 4(a), corresponding to ∼9  μs, resulting in an astonishing acceleration rate of $∼10^{6}\;m/s^{2}$. This sudden acceleration imparts a significant pressure impulse on the liquid at the nozzle, leading to the formation of the jet, which achieves a speed of ∼12  m/s at the same timescale. It is worth noting that the acceleration imparted by the bubble increases rapidly within 9  μs and then decreases exponentially (fit to an exponential decay function) with a time constant of ∼5.6  μs. The subsequent jet dynamics is driven by liquid inertia and bubble dynamics. The jet reaches its maximum speed when the bubble reaches its maximum diameter, at which point the bubble speed approaches zero.

As the bubble begins to collapse, a reentrant pressure appears near the nozzle exit, pulling the liquid inside the cavity while its upper part is still moving inertially, causing the jet to rupture. The pressure pulse and the meniscus at the nozzle resulted in a focused jet, and the subsequent bubble dynamics resulted in the ejection of the body of the jet. These findings are consistent with similar pressure impulses that have been reported using short-pulsed lasers and successfully numerically simulated, as described in the literature.^50^

### 4.3 Ejected Dose and Jet Power

**Fig. 5:**
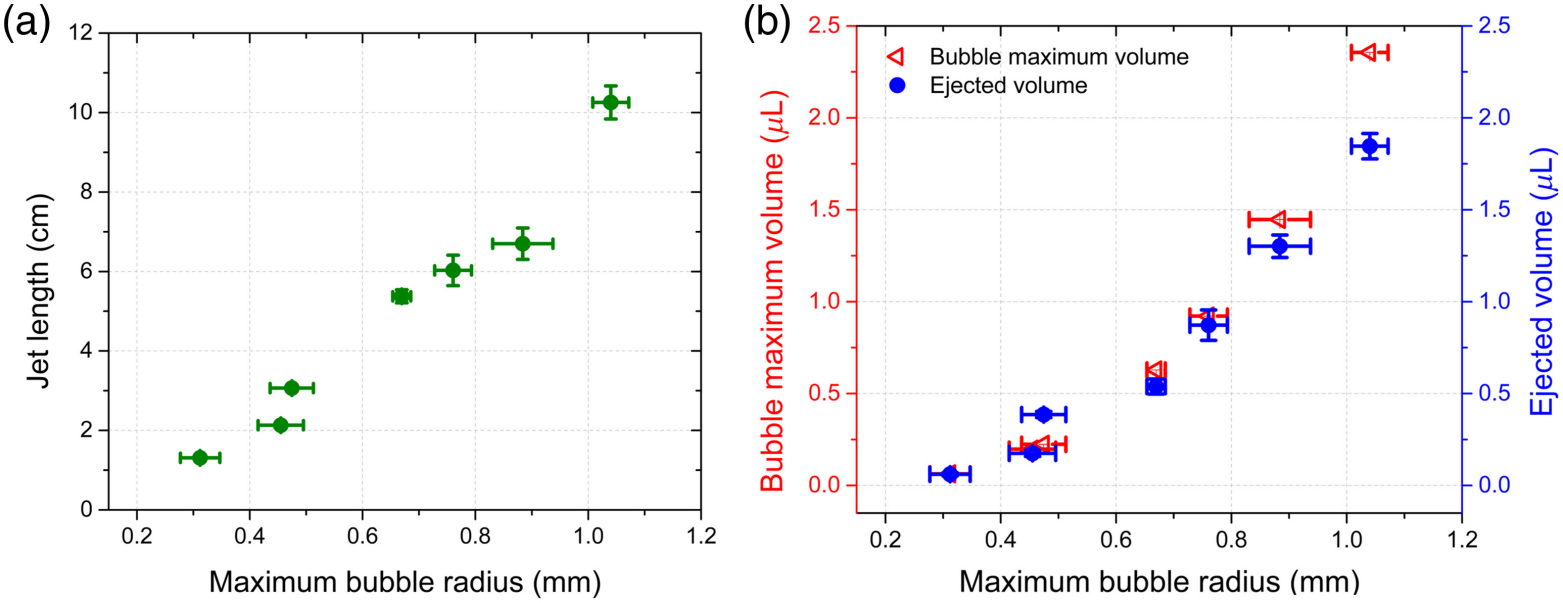
(a) Length of the liquid jets and (b) ejected volume per shot as function of the maximum bubble radius. Optical power of 1 W.

Figure 5(a) shows the jet length before breakup as a function of the beam spot size. The shortest jets (∼1.7  cm) are obtained from the smallest bubbles and the longest jets (10 cm) are obtained from the largest bubbles. Figure 5(b) shows that the volume of the ejected jets can be estimated from the length and the diameter of the jet, these measurements give a good estimate of the ejected volume, which varies from ∼0.1 to 2  μl. In addition, using the empty space after the bubble collapse, as shown in frame 30 of Fig. 3(a), a better estimate of the ejected volume can be obtained, which gives very close values to the previously determined volume. This means that in our device, the expelled volume can be effectively controlled from ∼0.1 to ∼2  μl simply by changing the focal position inside the chamber. In terms of volume expelled, our injector is far behind electromechanical injectors, which can deliver up to a volume of 1 ml^51^ per shot. However, the delivery of small volume doses may have certain advantages with respect to the administration of some types of drugs, faster injection rate, greater drug dispersion depth, and no visible damage to the skin.^33^ Previous injectors based on thermocavitation have reported delivery volumes ranging from 1 to 100 nl,^25^^,^^33^ which is attributed to the small volume chamber. Compared to short-pulsed laser-based devices, the ejected fluid volume varies from 1 to 1000 nl^52^^,^^53^ with the smallest volume achieved by the fastest jets (850 m/s) reported to date; however, one serious drawback of such high-speed jets is their small diameter, which is usually well below (typically one-tenth) the capillary diameter (100 to 500  μm).^16^^,^^24^^,^^54^^,^^55^

Dose is an important factor in the administration of drugs and vaccines. In the specific case of vaccines, the typical dose is in the range of 0.5 to 3 ml.^56^ In our study, the maximum volume of a single injection is ∼2  μl, so between 250 and 500 injections must be given before the vaccine dose is achieved. It is important to note that thermocavitation is a quasi-periodic phenomenon with a frequency in the kHz range.^22^ In principle, it would only take a few seconds to deliver the required dose. Our device delivers up to 20 jets of good quality before refilling. However, to ensure focused jets, continuous refill with an infusion pump instead of a syringe is required to preserve the meniscus.

When a jet of liquid moving at high speed strikes a solid surface, the impact pressure developed can be very high indeed, capable of permanently deforming or fracturing almost any high-strength structural material. This pressure is the result of the water hammer effect. The water hammer pressure $P_{\mathrm{WH}}$ for a flat-tipped liquid jet striking a rigid surface is given by^57^^,^^58^

$$P_{\mathrm{WH}}=\frac{\rho_{1}C_{1}\rho_{2}C_{2}}{\rho_{1}C_{1}+\rho_{2}C_{2}}V,$$ (1)

where $\rho_{1,2}$ is the liquid (substrate) density, $C_{1,2}$ is the speed of the sound in the liquid (substrate), and V is the liquid jet speed at impact. Since the agar content is small on our phantoms, the density and speed of sound are very similar to that of water, then Eq. (1) can be approximated to

$$P_{\mathrm{WH}}\approx \frac{1}{2}\rho_{1}C_{1}V.$$ (2)

**Fig. 6:**
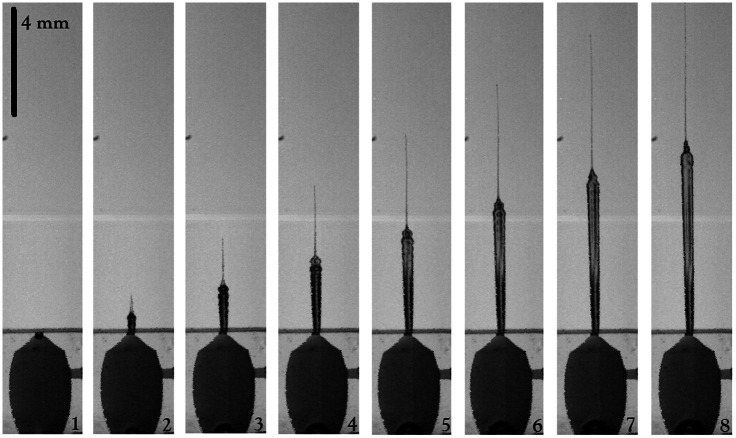
Typical jet shape obtained by hydrodynamic focusing. The liquid jet is generated with a laser power of ∼1  W and spot size of $\omega_{z}=346.9\;\mu m$ (z=23  mm). Each frame is acquired 37  μs apart. Maximum bubble radius ∼426  μm. Image scale bar is 4 mm.

**Fig. 7:**
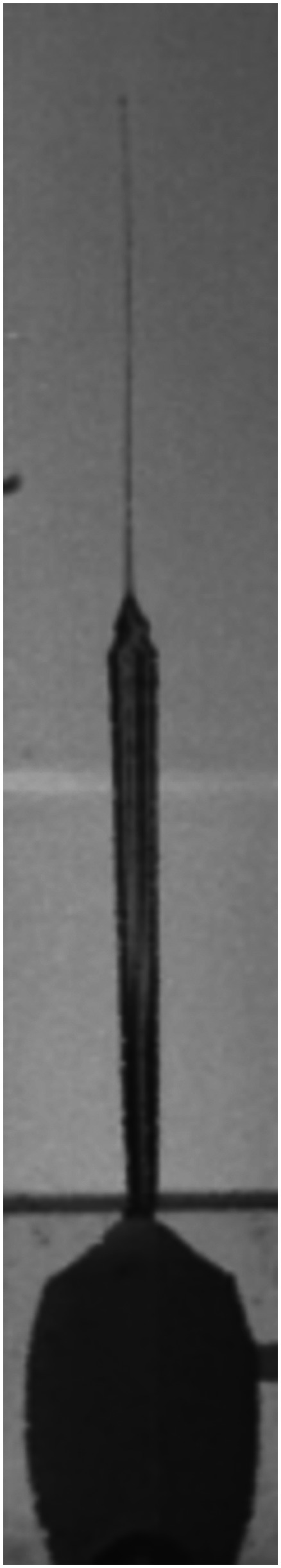
The bubble expansion drives the liquid jet out of the chamber. The finer jet is due to hydrodynamic focusing. Optical power 590 mW and $\omega_{z}=346.9\;\mu m$ (z=23  mm) (Video 1, MP4, 2.86 MB [URL: https://doi.org/10.1117/1.JBO.28.7.075004.s1]).

For drug delivery with needle-free injectors, the jet must break the stratum corneum, the outermost layer of the epidermis. The skin disruption pressure was reported ∼15 to 20 MPa^30^ and assuming a skin density of $1.15\;g/m^{3}$ and the sound velocity in the skin of 1730 m/s, the threshold speed can be estimated from Eq. (2) to be ∼7 to 10  m/s.^24^ From the speed of our jets, the exerted pressure ranges from 95 to 130 MPa, which is at least 6 to 8 times larger than the threshold pressure to break the stratum corneum. This pressure is exerted on a very short time (2 to 3  μs) even if the liquid is still imping on the substrate on. Thus, any jet with a pressure >15 to 20 MPa will certainly deliver drug through the skin. The water hammer pressure was used as parameter to indicate the breaking of the substrate^58^^,^^59^; however, in Eq. (2), there is no information on the jet diameter, this would imply that, for example, a jet with a diameter of 1  μm or 1 mm having the same speed will break the skin. To address this issue, the power of the microjet at the impact contains information on the density, diameter, and speed of the jets has been introduced to compare microjets of different sizes and speeds. Besides, the jet power is strongly correlated with the jet penetration [as shown in Fig. 9(a)] and the percentage of volume delivered.^60^ The power of a jet at the nozzle exit is related to the nozzle diameter, D, and the exit velocity, $V_{\text{jet}}$ is given by^29^^,^^54^^,^^60^^–^^63^

$$P_{\mathrm{jet}}=\frac{\pi \rho D^{2}V_{\mathrm{jet}}^{3}}{8},$$ (3)

where ρ is the fluid density for the saturated solution of $\mathrm{Cu}({\mathrm{NO}}_{3}{)}_{2}$ is $2000\;\mathrm{kg}/m^{3}$. Thus, the minimum power to just break the stratum corneum assuming a nozzle diameter of 200  μm and a jet of speed of 10 m/s is ∼30  mW. Equation (3) assumes that the diameter of the jet is equal to the diameter of the nozzle, which is not true in most cases. We note that completely filling up the chamber produces slower jets with a matchstick shape but when the chamber is filled just below the nozzle channel level, a meniscus is formed, changing the shape of the jet drastically. In fact, the quality and velocity of the jets depend critically on the formation of the meniscus. The resulting jets are characterized by a finer jet (tip) followed by a thicker jet (body). Figure 6 shows a typical jet obtained with an optical power of 1 W, and a beam spot of 346.9 mm (z=23  mm). For more information see Video 1 in Fig. 7. As will be shown below, the finer jet facilitates its penetration into agar o skin.

**Fig. 8:**
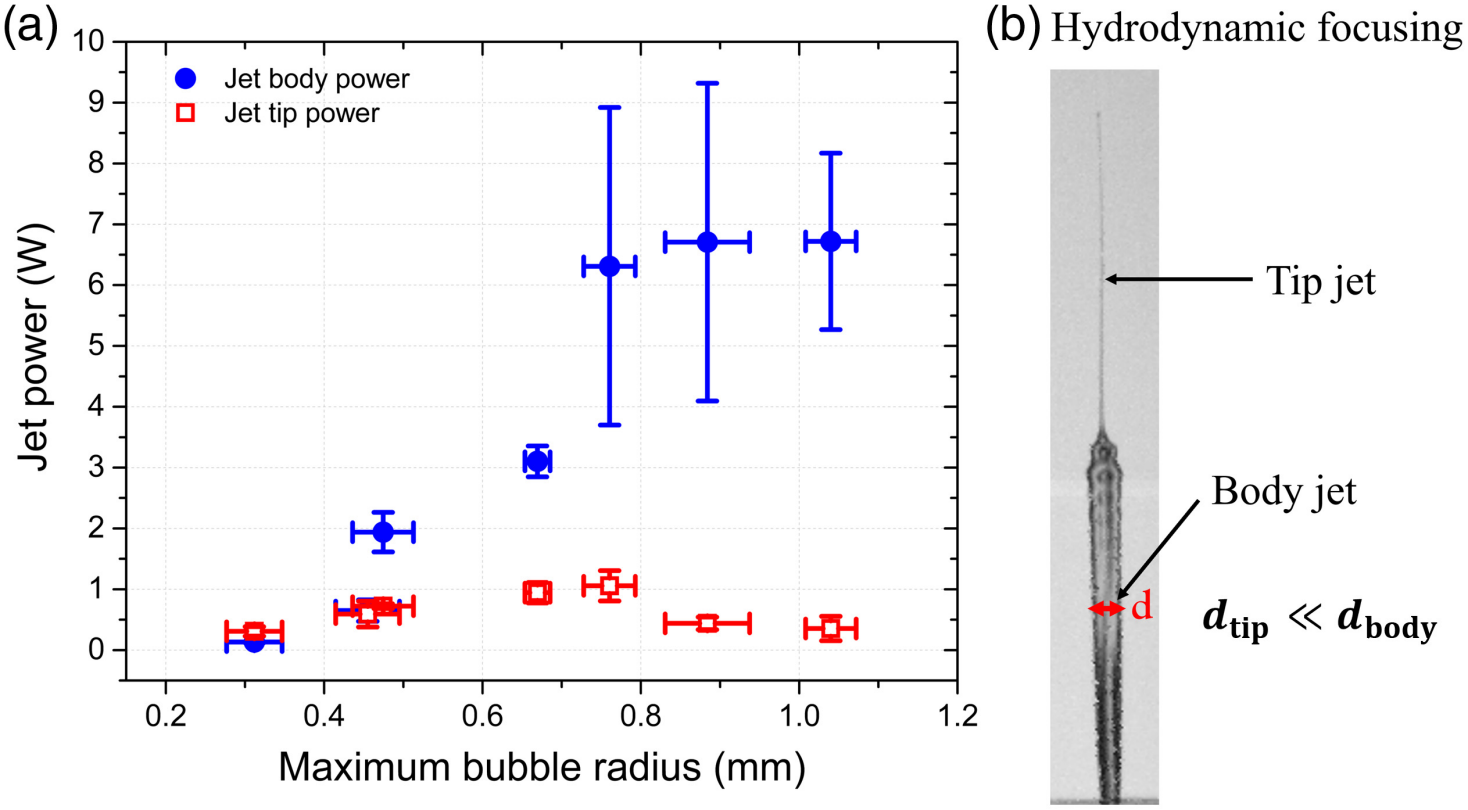
(a) Jet tip average diameter of 65  μm and jet body average diameter of 400  μm. Power of the tip and main body of the jet as a function of maximum bubble radius. (b) Hydrodynamic focusing produces jets with a thinner tip followed by a thicker jet (body). The liquid jet is generated with a laser power of ∼1  W and spot size of $\omega_{z}=346.9\;\mu m$ (z=23  mm).

Figure 8 shows the jet power for the tip and the body of the jet. The speed of the tip is about twice that of the body; the average diameter of the tip is ∼65  μm while the average diameter of the body is ∼400  μm. Given the small diameter of the tip, its power barely reaches 1 W, but it is powerful enough to break the stratum corneum at the highest speed while the body´s power reaches 6.7 W. It is worth mentioning that Eq. (3) does not hold for focused jets because the jet diameter is not constant at the nozzle. Nevertheless, we use this expression for our jets to get a rough estimate of the tip power. The exact value of the jet power requires a more complex analysis that takes into account the focusing process inside the nozzle, but such a study is beyond the scope of this work. The tip jet plays a critical role in the breaking of the skin as was demonstrated by Tagawa et al.^24^ Our results emphasize the importance of the diameter and velocity of the jets on the mechanical power. The impact power of our jets is well below the power obtained with pulsed laser-based devices (∼700  W).^54^ For comparison, the typical power of electromechanical injectors reaches up to ∼25  W.^61^ In fact, it has been shown that the shape of the jet determines the penetration depth: a lower power but collimated jet can penetrate deeper into porcine tissue than a higher power jet but with a dispersed shape.^64^ Thus, the special shape of our jets (thanks to hydrodynamic focusing) allows to break and penetrate into agar-based skin phantoms and *ex-vivo* porcine skin.

### 4.4 Jet Penetration in Agar Skin Phantoms and *Ex-Vivo* Porcine Skin

**Fig. 9:**
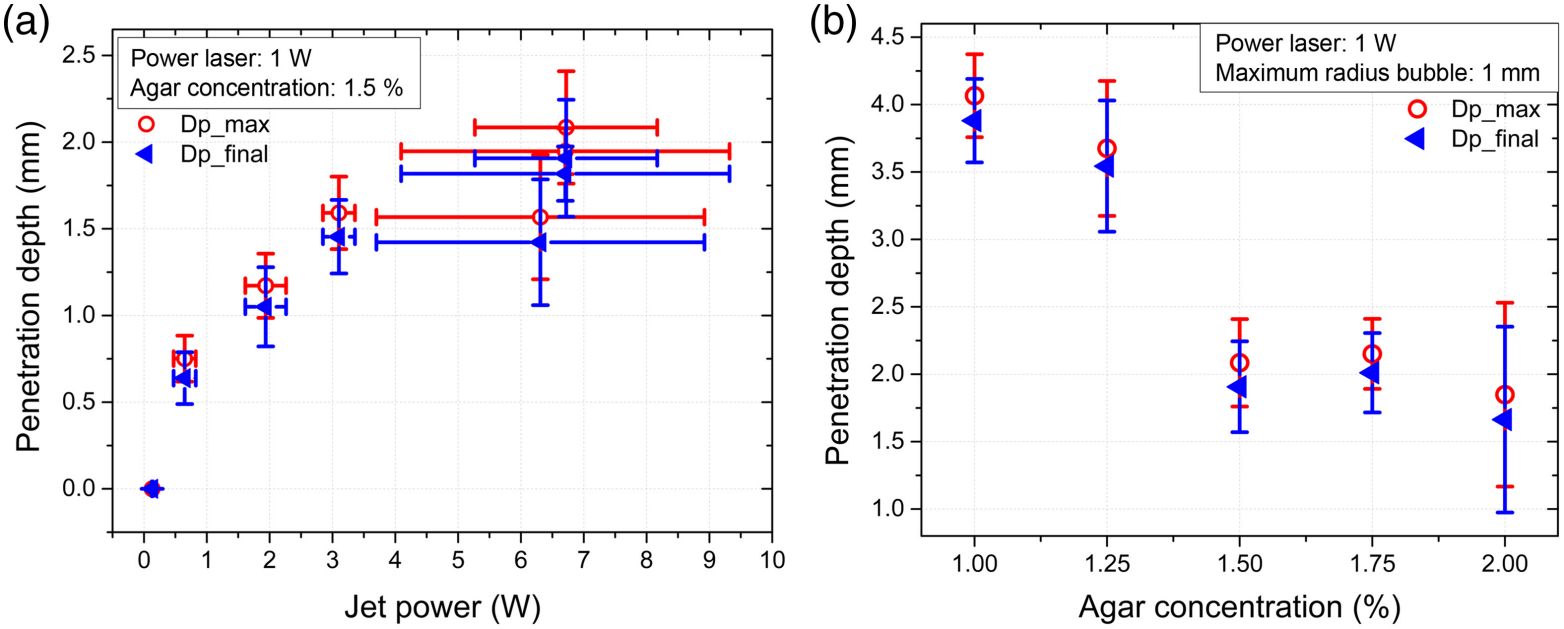
Penetration tests of liquid jets in agar gel. (a) Penetration distance of the liquid jet into agar gel with a concentration of 1.5% as a function of the jet power, using a laser power of about 1 W. (b) Penetration distance of the jets as a function of the agar concentration: 1%, 1.25%, 1.5%, 1.75%, and 2%.

**Fig. 10:**
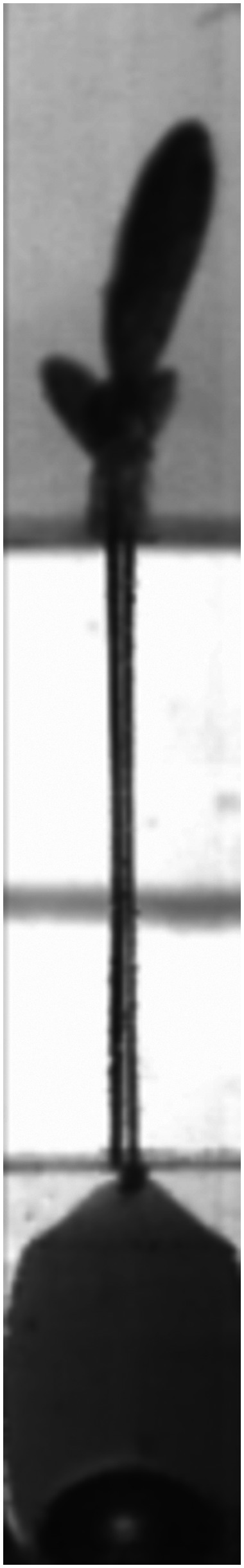
Jet penetration into a phantom with 1% agar concentration. The phantom is placed 5 mm away from the chamber. Optical power 1 W and $\omega_{z}=512.3\;\mu m$ (z=34  mm). (Video 2, MP4, 1.98 MB [URL: https://doi.org/10.1117/1.JBO.28.7.075004.s2]).

Figure 9(a) shows the penetration depth in 1.5% agar gel versus jet power. The penetration depth increases with the jet power in agreement with previously published results. Using the Pearson correlation method, a correlation coefficient of ∼0.91 (∼91%) and a P-value of 0.0045 (<0.05) were obtained. This indicates a strong correlation between jet penetration depth and jet power. The jet penetrates the agar to a maximum penetration length ($D_{p-\max}$), but since the agar is an elastic medium, some liquid will be expelled after it returns to its original shape. The final penetration length is $D_{p\_\mathrm{final}}$ is ∼90% of $D_{p-\max}$. The optimal conditions to obtain the most powerful and fastest jets are optical power 1 W, ∼70  m/s average velocity, laser spot size $\omega_{z}=512.3\;\mu m$, z=34  mm, and average power of ∼7  W. Figure 9(b) shows the penetration length in skin phantoms with different agar concentrations. Obviously, the largest average penetration (∼4  mm) was obtained for the phantom with the lowest agar concentration (1%) and the smallest average penetration (∼1.5  mm) was obtained for the highest agar concentration (2%). Figure 10 shows a video of the jet penetration into 1% agar skin phantom. The standoff distance, i.e., the distance between the nozzle and the target, was varied from 1 to 9 mm and an average penetration of 2 mm was obtained in the 1.5% concentration with the lowest intensity, i.e., the depth penetration is approximately independent of the standoff distance. Note that the large variation in penetration depth in all experiments is mainly due to the variation in the bubble diameter of the thermocavitation bubbles. These results, indicates that our jets could easily penetrate into hypodermis, muscle and fat (elastic modulus of 1 to 20 kPa), dermis and full thickness skin (20 to 100 kPa), and stratum corneum (100 to 500 kPa).^54^

**Fig. 11:**
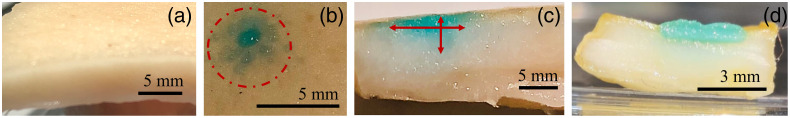
(a) *Ex-vivo* fresh porcine skin. (b) View of the porcine skin area after the injection of the liquid jet (red circle). (c) Transversal section to the porcine skin showing lateral diffusion of the liquid. (d) The solution was applied topically to the skin after 1 h. The blue color is due to copper nitrate.

To prove the latest assertion, fresh *ex-vivo* porcine skin was obtained from a local butcher and cut into cubes of 1.5 cm side length. Jets of ∼7  W were directed at the skin, which was placed at a distance of 5 mm. Immediately after injection, the remaining solution on the skin was removed to prevent diffusion into the skin. Figures 11(a) and 11(b) show the *ex-vivo* porcine skin before and after the injection. After the injection, a transversal section [Fig. 11(c)] was made to visualize the liquid penetration, which is ∼3 to 4 mm and diffuses laterally almost ∼5 to 7 mm. This pattern is quite different from the pattern in agar indicating the difference in the constitutive nature of agar and skin as reported previously.^5^^,^^32^^,^^33^^,^^55^^,^^65^ In addition, a drop of the solution was applied topically to the skin for several minutes up to 1 h. It was found that diffusion into the skin is a very slow process (even after 1 h of topical application) with a penetration depth into the skin [Fig. 11(d)] smaller than that obtained with the injected solution. Note that the skin swallows possibly due to the corrosive nature of copper nitrate. A detailed study of jet penetration in *ex-vivo* porcine skin is required but is beyond the scope of this paper.

The use of copper nitrate solution as a light-absorbing material is a perfect candidate to demonstrate the working principle and capability of thermocavitation-based injectors. However, copper nitrate is a toxic and corrosive solution, so a non-toxic one must be found to more accurately determine the depth penetration and drug diffusion extension in the skin. The most viable option in thermocavitation-based injectors is to divide the chamber into two compartments separated by an elastic and impermeable membrane.^30^^,^^32^ One chamber contains the solution where thermocavitation occurs, whereas the second contains the drug to be injected. This minimizes thermal damage to the drug. Copper nitrate could be replaced by pure water, but this requires the use of a laser emitting at 1.9∼3  μm, where the water absorption coefficient is greatest.^66^^–^^68^ Finally, the use of a metallic thin film, such as titanium, deposited on the bottom substrate is also a good option if lasers emitting in the visible part of the spectrum are used.^69^

### 4.5 Comparison between Thermocavition Generated Jets and Other Mechanisms

Table 1 shows a comparison of the performance of our device with other needle-free injectors of competing technologies i.e. short & long-pulsed laser, commercial and thermocavitation-based devices. Long-pulsed lasers (hundreds of microseconds) refer to lasers operating at 2 to 3  μm wavelength where the water absorption is high. Bubbles produced with these lasers are mistakenly attributed to multi-photon absorption, but the mechanism is most likely a single-photon one, i.e. thermocavitation. As can be seen, there is a wide range of parameter variation, for example, the jet power ranges from 25 to 1000 W. The power of commercial devices is in the range of 100 to 200 W, the power of short-pulse based devices is in the range of 200 to 1000 W (mainly because of its high speed due to the hydrodynamic focusing), whereas thermocavitation devices produce the least powerful jets (<10  W). The fastest jets are achieved using pulsed lasers in capillary tubes. Commercial devices achieve speeds between 100-200 m/s while thermocavitation devices barely reach 100 m/s. According to our results, the speed of the jets can be optimized by a clever design of the cavity. In addition, an interesting approach to increase jet speed is the use of momentum’s transfer of the pressure waves (emitted by the bubble collapse) at the liquid-air interface.^22^^,^^25^ In terms of jet penetration, comparison among the competing technologies is not easy as different materials have been used to fabricate skin phantoms or even different types of skins have been used. Nevertheless, our device is competitive, although it does not achieve the highest power and speed, but the special shape of the jets provides a competitive advantage.

**Table 1:** Comparison of the performance of our device with other needle-free injectors of competing technologies (some power values were calculated from data extracted from the paper).

Reference	Device	Technology	Jet speed (m/s)	Power (W)	Penetration depth (mm)	Sample
70	Capillary tube	Short pulsed laser	320 (∼170)	11.58 (1.73)	0.5 (5)	Gelatin 5%
71	Capillary tube	Short pulsed laser	250 (140)	15.34 (2.7)	1.5 (∼2.3)	Skin from the back of a rat (Gel 5%)
54	Capillary tube	Short pulsed laser	605 (1072)	19.8 (1088)	1 (0.1)	Hydrogel (69.5 kPa, 462 kPa)
16	Chamber	Short pulsed laser	230	47.77	1.2	Gelatin 5%
53	Chamber	Long pulsed laser	120.5	15.46	2.3 to 3.4 (0.35)	Polyacrylamide gel 10%–30% (60 to 380 kPa) (Porcine skin)
63	Chamber	Long pulsed laser	160	36.2	0.4	Porcine skin
32	Chamber	Long pulsed laser	140	24.24		Porcine skin (10 to 30 MPa)
23	Chamber	Thermocavitation	94	9.4	1	Agarose gel 1% (40 kPa)
72	Chamber	Thermocavitation	48	0.63	1.3	Agarose gel 1%
73	Chamber	Thermocavitation	25	0.061	0.4	Pig skin
**This work**	**Chamber**	**Thermocavitation**	**65 (28.7)**	**0.32 (6.63)**	**4 to 1.85**	**Agarose gel 1% to 2% (34 to 113 kPa)**
74		Piezo actuator	180	5.72	0.5 to 9	Polyacrylamide gel 4% to 30 % (10 to 500 kPa)
62		Spring	142	27.2	30 to 60	Gelatin 4, 5 y 10% (42.61 to 906.9 kPa)
75		Lorentz force	200	152	13 to 17	Acrylamide gel 10% to 20% (60 to 240 kPa)
61		Compressed gas	134	12.28	16 to 19.3	Gelatin 2%, 5%, and 8%
51		Pneumatic pressure	180	22.9	8.9	Muscle in cadavers (15 kPa)

**Fig. 12:**
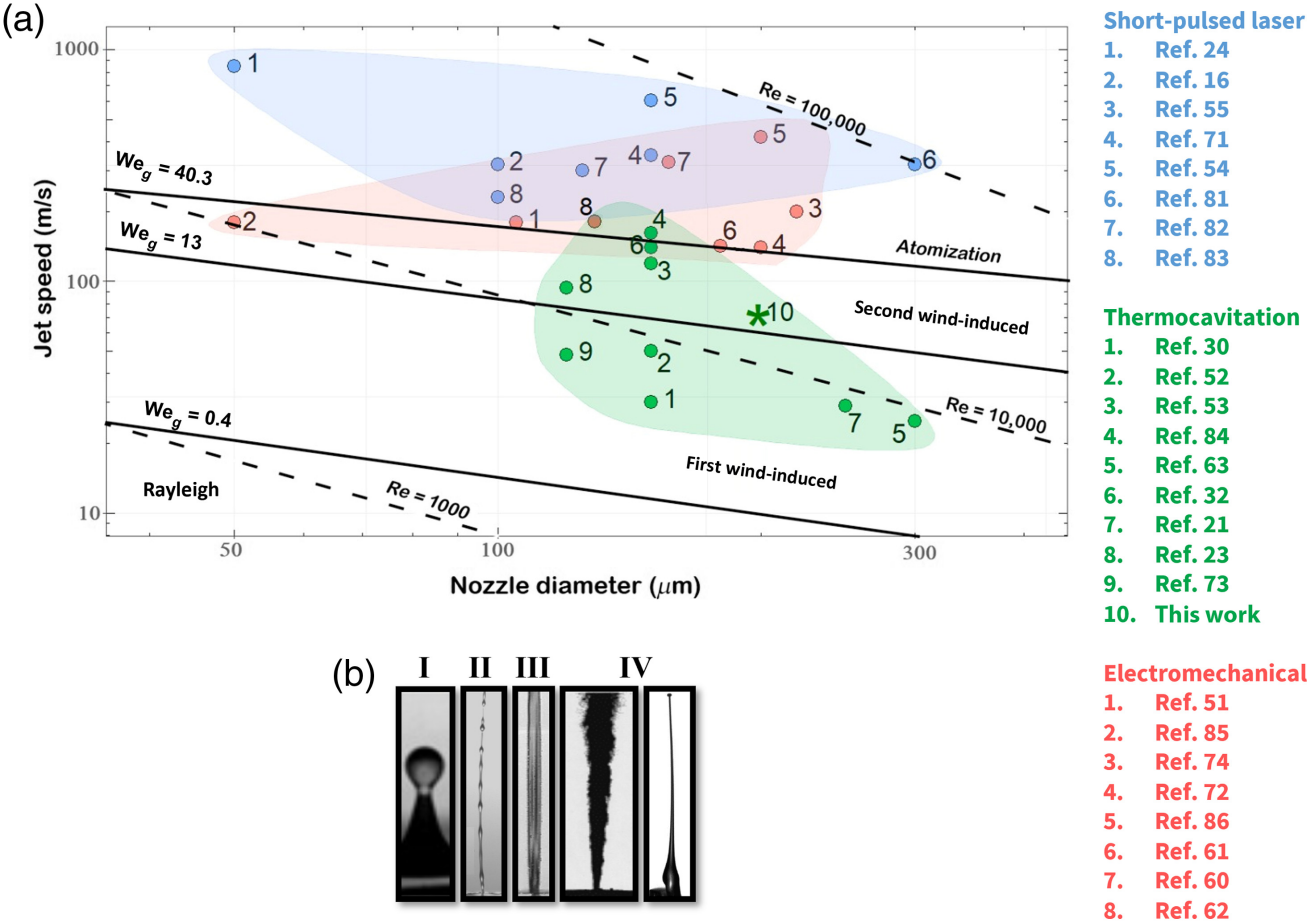
(a) Map of jet breakup regimes.^16^^,^^21^^,^^23^^,^^24^^,^^30^^,^^32^^,^^51^^–^^55^^,^^61^^–^^63^^,^^71^^,^^72^^,^^74^^,^^75^^,^^80^^–^^86^ The asterisk symbol represents the results presented in this paper. (b) Typical jet shape for the different breakup regimes. Figure I was adapted from Ref. 79, and Fig. IV is adapted from Refs. 69 and 24.

Given the wide range of jet speeds reported in Table 1, it is natural to ask if there is an upper limit to jet speed. When a liquid jet is ejected, perturbations occur at the jet surface because of the competition between cohesive and disruptive forces. Despite the complex nature of the jet dynamics, the linear stability theory can provide qualitative descriptions of breakup phenomena and predict the existence of different breakup regimes.^69^^,^^76^^–^^79^ For the steady injection of a liquid through a single nozzle with a circular orifice into air, the jet breakup mechanisms are typically classified into four main regimes (Fig. 12) according to the relative importance of inertial, surface tension, viscous, and aerodynamic forces. Each regime is characterized by the magnitude of the Reynolds number Re (which expresses the ratio of inertial to viscous forces), the aerodynamic Weber number ${\mathrm{We}}_{g}$ (which is the ratio between the deforming inertial force and stabilizing cohesive force), and the Ohnesorge number Oh (which relates the viscous to inertial and surface tension force)^87^

Re=ρLVjetL/μL, (4)

$${\mathrm{We}}_{g}=\frac{V_{\mathrm{jet}}^{2}\rho_{L}L}{\sigma },$$ (5)

Oh=WegRe=μLρLσd0, (6)

where $\mu_{L}$, σ, $\rho_{g}$, $\rho_{L}$, and L are the dynamic viscosity of the liquid, surface tension, air density, liquid density, and the characteristic distance (nozzle/jet diameter), respectively. Reynolds numbers below $∼10^{4}$ indicate laminar flow while at Re>104 the flow becomes turbulent. The aerodynamic Weber number describes the instability in the jet when the aerodynamic forces become significant. Finally, the Ohnesorge number is used to study the dispersion of liquids in gases and in spray technology. Depending on the jet speed, the geometrical factors of the nozzle, and the rheological properties of the flows, the breakup of a liquid jet in a quiescent gas can occur in the following four regimes:^76^^,^^77^ (a) Rayleigh breakup, (b) first wind-induced breakup, (c) second wind-induced breakup, and (d) atomization.

Figure 12 shows the operating scheme for the different injector technologies, divided into three groups: commercial electromechanical methods (red numbers), short-pulsed optical cavitation (blue numbers), and CW optical cavitation (thermocavitation, green numbers). For needle-free injector applications, typical nozzle diameters are in the range of 100 to 500  μm and the speed varies between ∼20 to 1000m/s. The different regimes described above are separated by a solid line indicating a Weber number. The dashed line indicates the Rayleigh number, so the Rayleigh number for jet injectors lies between $10^{4}$ and $10^{5}$. The first wind-induced regime is reached when the surrounding gas inertial force reaches 10% of the surface tension force (${\mathrm{We}}_{g}<0.4$). In the second wind-induced regime, the interaction with the surrounding gas begins to dominate over the other forces. The limits for this regime are associated with a certain value of the aerodynamic Weber number ($13<{\mathrm{We}}_{g}<40$). Finally, ${\mathrm{We}}_{g}>40$ are typical of the atomization regime. The second wind-induced breakup and atomization regime are of particular interest for needle-free injectors because the jet can break the stratum corneum. Figure 12(a) shows that commercial injectors are on the boundary between the second wind-induced breakup regime and the atomization regime or well within the former. It is therefore easy to understand why they are unstable and usually form a spray. Interestingly, the jets speed produced by pulsed lasers, although, well within the atomization regime they do not break up possibly because the hydrodynamic focusing avoids contact between the liquid and the nozzle walls preventing the formation of cavitation bubbles that might otherwise disturb the jet. Thermocavitation, probably produces the most stable jets as they are in on the second-wind induced regime. Figure 12(b) shows typical jets corresponding to the four different regimes.

## 5 Conclusions

We have demonstrated that high-speed jets can be obtained from thermocavitation bubbles inside a chamber carved in transparent glass. Thanks to the innovative fabrication technique—FLICE—it was possible to fabricate a large monolithic chamber, avoiding joints and bonding, with obvious advantages in robustness, no leakage, and resistance to high pressure. A high-speed camera was used to study the bubble and jet dynamics. It was found that the maximum bubble wall speed is ∼10 to 25 m/s for almost any combination of laser parameters. This means that the velocity of the liquid jets produced by thermocavitation is limited by the bubble dynamics, but a proper cavity design can produce jets with an average speed of 70 m/s. The ejected volume can be controlled from ∼0.1 to ∼2  μl simply by changing the focal position within the chamber. The volume delivered by our injector is much lower than electromechanical injectors, which can deliver up to 1 ml per shot. However, the delivery of small-volume doses may have certain advantages in terms of the administration of some types of drugs, faster injection rate, higher drug dispersion depth, and no visible damage to the skin. Compared to short-pulsed laser-based devices, the volume of fluid ejected varies from 1 to 1000 nl, with the smallest volume achieved by the fastest jets reported to date (850 m/s); however, a serious drawback of such high-speed jets is their small diameter, usually well below (typically one-tenth) the nozzle diameter. The quality and velocity of the jets depend critically on the formation of a meniscus to provide hydrodynamic focusing. The resulting jets are characterized by a finer jet (tip) followed by a thicker jet (body). These jets penetrate up to 1 mm (on average) in the hardest agar (2%) and 2 mm in *ex-vivo* porcine skin. In summary, our research suggests that needle-free injectors based on thermocavitation have the potential for significant commercial development, particularly with the utilization of low-cost, fiber-coupled lasers. However, further research is necessary to optimize the technology and ensure its safety and efficacy for widespread use. As with any new medical technology, cautious and thorough evaluation is essential before considering its commercialization.
